# Lithium Treatment Over the Lifespan in Bipolar Disorders

**DOI:** 10.3389/fpsyt.2020.00377

**Published:** 2020-05-07

**Authors:** Constantin Volkmann, Tom Bschor, Stephan Köhler

**Affiliations:** ^1^ Department of Psychiatry and Psychotherapy, Charité – Universitätsmedizin Berlin, Berlin, Germany; ^2^ Department of Psychiatry, Schlosspark Hospital Berlin, Germany; ^3^ Department of Psychiatry and Psychotherapy, University Hospital, Technical University of Dresden, Dresden, Germany

**Keywords:** bipolar disorder, lithium, efficacy, lifespan, suicidality, discontinuation

## Abstract

Lithium has been the treatment of choice for patients with bipolar disorder (BD) for nearly 70 years. It is recommended by all relevant guidelines as a first-line treatment for maintenance therapy. In this review, we outline the current state of evidence for lithium in the treatment of BD over the lifespan. First, we summarize the evidence on efficacy in general, from relapse prevention to acute anti-manic treatment and its role in treating mood episodes with mixed features and bipolar depression. As patients are often treated for many years and different aspects have to be considered in different phases of life, we discuss the particularities of lithium in the treatment of paediatric BD, in older aged individuals and in pregnant women. Lastly, we discuss the evidence on lithium's proposed suicide-preventive effects, the dangers of rapid discontinuation and lithium's adverse effects, particularly with regard to long-term treatment.

## Background

Bipolar disorder (BD) is an episodic illness with a very heterogeneous clinical course. It usually presents as a severe, chronic, and disabling condition characterized by mood alterations between euthymia, major depression, and (hypo-)mania. The estimated lifetime prevalence ranges from 0.6% to 2.4% worldwide (1, 2). BD is usually a lifelong disease, hence requiring lifelong treatment strategies. One of the major pharmacological agents in the treatment of BD is lithium. It remains the gold standard in preventing recurrences in BD I (mania and depressive episodes) and BD II (hypomania and depressive episodes) and is effective in the treatment of mania. Additionally, the proposed anti-suicide effect of lithium is unique and potentially of high relevance in the treatment of BD over the lifespan, as patients with BD suffer from high suicide rates (3). Over the last decades, other substances such as second generation antipsychotics (SGA) and anticonvulsants have been prescribed more frequently and there has been a tendency to avoid lithium in the treatment of BD. Reasons may be the overestimation of potential side effects as compared to other substances by professionals and patients alike, despite the highly problematic metabolic profile of antipsychotics (e.g., Olanzapine), particularly over the lifespan (4).

This narrative review focuses on lithium-treatment over the lifespan in BD and gives a summary of its effectiveness, side effects, and treatment recommendations with regard to specific treatment conditions and subtypes of BD. Furthermore, we discuss the risk of lithium-discontinuation, which is an important topic in the treatment of BD over the lifespan.

## Efficacy of Lithium in BD

The pharmacological treatment of BD has several goals. Lithium is the agent of the “first hour” in the treatment of BD and has been used over decades in all phases of the disease. Lithium treatment aims at the prevention of relapses and is used in the treatment of acute episodes, such as mania, depression, and specific subtypes, such as mood episodes with mixed features or rapid cycling (RC) (see below). Especially with regard to the lifespan, the efficacy of lithium in special treatment conditions, such as BD in paediatric and older aged patients and BD during pregnancy and postpartum are of high relevance. For each of these conditions, different recommendations are available from different treatment guidelines (5). The German S3 guidelines also recommends lithium as the first choice of treatment for patients with high risk for suicidality (6). First, we give a short summary of the general effects of lithium in mania, depression, and maintenance treatment of BD.

### Lithium in Maintenance Treatment

Preventing new episodes in BD is essential with regard to quality of life, participation in society and preventing long-term disability. Lithium remains the gold standard in achieving this goal. It is effective in both type I and type II BD (e.g., (5)). Only for lithium, randomized controlled trials (RCTs) without an “enriched design”, favoring the substance to be investigated, have been performed (7, 8). Several meta-analyses of randomized placebo-controlled, long-term trials could verify that lithium significantly reduces the risk of new episodes (mania and depression) compared to placebo (e.g., (9–12)). A Cochrane review found the risk of any relapse to be 36% for lithium and 61% for Placebo over the course of 1 year, corresponding to an absolute risk reduction of 25% [NNT = 4, (13)]. Kessing et al. as well as Hayes et al. found lithium monotherapy to be superior to monotherapy with other maintenance mood stabilizers in real life conditions (14, 15). This is reflected by its status as the first-line drug in many international guidelines (5). For optimal efficacy in long-term treatment of BD, target serum levels should generally be around 0.6–0.8 mmol/L, while specific treatment situations and patients may require slightly higher or lower lithium levels (16).

### Lithium in Treatment of Mania

While manic episodes are often the most impressive part of BD, their duration is usually shorter compared to that of other disease phases in both BD-I and BD-II. A number of pharmacological agents have been investigated and have proven their efficacy in the treatment of mania, including lithium (17). The network meta-analysis by Cipriani et al. found an effect size of 0.40 SMD, indicating a moderately sized effect, which is comparable to other anti-manic agents (18). For example, lithium showed a comparable efficacy to quetiapine in a 12-week RCT (19). Despite its proven anti-manic properties, lithium has lost some of its relevance in the treatment of mania in the recent years, partly due to the large number of SGAs that have been licensed for this indication. Reasons for favoring SGAs over lithium include the shorter time of dosage increase and sedation, a common side effect of SGAs that is often welcomed during manic agitation. Further potential disadvantages of lithium include the small therapeutic window and hence the necessity for repeated serum level controls (20). In addition, no parenteral application is available for lithium. Notwithstanding these limitations, lithium should be considered as a first-line therapy for manic episodes, as long-term phase-prophylactic treatment is almost always required afterward, for which lithium is considered the first choice. In the treatment of manic episodes, higher levels of 0.8 to 1.2 mmol/L are required in order to achieve optimal response.

### Lithium in Treatment of Bipolar Depression

Bipolar depression is the predominant pole in BD type I and type II and responsible for a large number of suicides. The suicide rate is 20-times above that of the general-population (21), which is considerably larger than that of unipolar depression (22). Bipolar depression is also associated with a high rate of morbidity and mortality due to comorbid somatic disorders (23). However, treatment of bipolar depression is challenging for clinicians, as the classical treatment strategies of unipolar depression (antidepressants, lithium) show small(er), if any, effects (24). The lack of effectiveness of antidepressants in BD has been the topic of an ongoing controversy (25, 26). There is evidence that antidepressants may worsen the course of the disease in patients with mixed symptoms or RC by increasing the switch risk or causing tachyphylaxis after repeated antidepressant drug exposures (27).

Similarly, the available data strongly doubt the effectiveness of lithium in treating bipolar depression. The large EMBOLDEN I study found that lithium was not more effective than placebo in treating bipolar depression (28). In an open-label RCT comparing venlafaxine and lithium in bipolar depression in BD type II, lithium was significantly less effective than the antidepressant (29). However, relatively low lithium serum levels are a possible limitation of this and several other trials investigating lithium in the treatment of bipolar depression. Albeit the lack of evidence supporting lithium monotherapy in the treatment of bipolar depression, there are substantial differences in international treatment guidelines with regard to the role of lithium in bipolar depression (30). Some guidelines, such as the CANMAT guidelines (31), still regard lithium as a first line treatment option in bipolar depression. These guidelines argue that lithium should be considered in the acute treatment of bipolar depression due to its central role as a mood stabilizer, its effectiveness in preventing mania and the proposed anti-suicide effect. These guidelines highlight the low number of studies investigating lithium in bipolar depression and the limitations of these studies. Contrarily, the German S3 guideline does not recommend the use of lithium monotherapy in the treatment of bipolar depression (6). The WFSBP guideline agrees with that recommendation and emphasizes the combination therapy of lithium with other agents in bipolar depression (32).

## Lithium in Episodes With Mixed Features and Rapid Cycling

BD, especially in the lifelong course, is typically characterized by recurring mood episodes of opposite polarity. However, patients may also experience episodes in which depressive and manic symptoms co-occur. These phases were traditionally called mixed states or mixed episodes. The DSM-5 substituted these terms by the so-called “mixed specifier”, which indicates the presence of mixed symptoms in either (hypo-)manic or depressive episodes (33). Patients with mixed features in the course of their illness have a considerably higher risk to commit suicide and higher rates of (psychiatric) comorbidities (e.g., anxiety disorders, substance dependence and personality disorders). They suffer from high rates of relapses and experience a larger number of new episodes compared to BD patients without mixed symptoms (34).

Treatment of patients suffering from affective episodes with mixed features is particularly demanding for clinicians, especially as there is a lack of RCTs investigating these patients. The available data on the effectiveness of lithium in mood episodes with mixed symptoms are inconclusive (33, 35). In patients with a manic episode and additional depressive symptoms, lithium was found to be less effective than valproate (36).

Studies investigating the effectiveness of lithium in the maintenance therapy found it to be less effective in patients with mixed symptoms than in patients with “pure” mania. However, episodes with mixed features may generally be more difficult to treat than classic episodes. A *post hoc* analysis of lithium in “dysphoric mania” even found lithium not to be more effective than placebo (37). However, there are several shortcomings of all of these studies, for example the post-doc design and limitations with regard to the patient population. Given the high rates of suicidality during episodes with mixed symptoms and the anti-suicide properties of lithium, it should not be ignored as a pharmacological treatment-option in these patients. However, (more) positive data (mainly based on *post hoc* analyses) are available for SGAs and valproate in the treatment of BD with mixed features.

Another clinical phenotype of BD is the concept of RC. RC is defined as a course of BD that includes at least four distinct mood episodes (including major depressive, manic, hypomanic, with or without mixed features) occurring within a 12-month period. RC has a lifetime prevalence of 5 to 33 % among BD patients, which emphasizes its clinical relevance (38, 39). Patients with RC suffer from a higher burden of symptoms, higher rates of suicide attempts and completed suicides and they are frequently suffering from other psychiatric comorbidities over the lifespan of BD (40, 41). Patients with RC show a prolonged and complex course of their disease and high rates of treatment failure compared to patients without RC (42). Risk factors that are associated with the onset of RC are female gender, hypothyroidism, and antidepressant medication (43).

Only very little data on the treatment of RC are available from randomized clinical trials. Naturalistic and non-controlled studies suggest that lithium is less effective in patients with RC than in those without (44). However, all available treatments show lower effectiveness in patients with RC and lithium may show better efficacy than other mood stabilizers except for valproate (45). In a re-analysis of patients with BP II receiving venlafaxine and lithium, however, there were no differences in response or relapse rates between patients with or without RC (46). In a study from Suppes et al., no differences could be found between lithium and lamotrigine in reducing depressive symptoms in patients with bipolar depression and RC (47). Regarding long-term treatment of BD and RC, lithium has been investigated in a prospective trial (cross-over design, duration of 2 years) in comparison to carbamazepine or the combination of both (48). Lithium monotherapy was inferior to the combination therapy in patients with a history of RC, while there was no difference to carbamazepine monotherapy. In addition, lithium monotherapy was equally effective in comparison to a combination treatment of lithium and valproate in RC (relapse rates) and in comparison to valproate monotherapy (49).

## Lithium in Paediatric Bipolar Disorders

BD often begins in adolescence (50) and usually requires lifelong treatment including pharmacotherapy and psychosocial interventions (51). It is important to distinguish between paediatric and adult BD when choosing the appropriate medication, as side effects may affect patients of different age groups differentially.

Lithium has been approved for the acute and maintenance treatment of mixed and manic episodes of BD I in children and adolescents (age from 7 to 17 years) by the FDA. The effectiveness of lithium in paediatric patients has been demonstrated by numerous studies [e.g., (52, 53)].

In young patients with BD, the risks and benefits of pharmacological (long-term) treatment have to be balanced even more carefully, as the longer duration of exposure to the medication poses particular risks of adverse events. Before starting lithium treatment, standard examinations such as baseline laboratory checks of thyroid hormones, creatinine, blood salts, and calcium levels should be performed. The monitoring of lithium-levels and of other blood parameters is comparable to that of adults. As in all BD patients but especially in paediatric BD, the spectrum of lithium adverse effects should be thoroughly discussed, especially with regard to lithium intoxication. In adolescents, adequate hydration during the summer and during high levels of physical activity are important concerns the patient should be informed of. Cases of significant dehydration (such as due to emesis or diarrhoea) can make a dose reduction or pausing of the medication necessary. Like in adults, thyroid hormone status has to be closely monitored, as lithium may lead to hypothyroidism requiring the substitution of thyroid hormones (54). In paediatric BD, acute and long-term treatment with lithium seems to have relatively little effect on body weight (53, 55) and may therefore be preferred to antipsychotics with their unfavorable metabolic profile. Serious side effects of lithium in paediatric BD, which have been reported and therefore must be recognized, are suicidal ideation, change of blood cell count, and neurological side effects (54).

### Dosing Strategies in Paediatric BD

The elimination half-time of lithium is significantly shorter and the clearance of lithium significantly higher in paediatric patients. Therefore, Landersdorfer et al. (56) recommend a twice-daily dosing of lithium to achieve acceptable blood concentrations. Data are missing for once-daily dosing of lithium in paediatric BD and is thus not recommended.

To summarize, lithium is an effective treatment strategy in the treatment of paediatric BD in different phases of the disease, in mixed and manic episodes and in the maintenance phase.

## Lithium Use in Pregnant and Postpartum Women With BD

Women with BD are at an elevated risk of recurrence during the peripartum and postpartum period with relapse rates as high as 40–70% (57, 58). Recent research suggests that lithium discontinuation may be responsible for the elevated relapse risk during pregnancy, as pregnancy itself only has a small or even no effect on relapse rates in BD (57, 59). A majority of pregnant women with BD decide to self-discontinue lithium or even have problems getting a prescription for lithium (60, 61). There is a high variability in the available information and recommendations regarding lithium treatment during pregnancy (62). While lithium is the most recommended agent in BD during pregnancy, there is a lack of high-quality data. Observational studies support the use of lithium in the postpartum period in relapse prevention (58). However, the benefits of relapse prevention have to be weighed against potential adverse effects on mother and child (63).

One major fear is the assumed teratogenicity of lithium during pregnancy, especially the risk of Ebstein's anomaly. In one of the largest cohort studies, Paterno et al. found that lithium intake during the first trimester is associated with an increased risk of cardiac malformations. In this study the adjusted risk ratio for cardiac malformations between exposed and non-exposed infants was 1.65 (95% confidence interval [CI], 1.02 to 2.68) and it showed a dose-dependent relationship. Nevertheless, the effect size was lower than previously assumed, with an absolute risk increase of 1.25% (64). In a meta-analysis by Munk-Olsen (65), lithium intake during pregnancy did not correlate with complications during pregnancy or delivery. However, the authors reported an increased risk for “cardiovascular defects, neural tube defects, hypospadias, and epispadias (OR 1.71), but not for major cardiac malformations (e.g., Ebstein's anomaly)”. For a detailed overview, we recommend the review of Hermann et al. (62).

Several experts recommend continuing lithium in patients with BD I who have been stable on lithium to prevent relapse or even mood instability. Nonetheless, a critical discussion should be held with the patient that involves the risks, benefits, and alternatives for treatment during pregnancy and postpartum. Changing of medication during pregnancy and postpartum should be avoided when possible (62). Patients continuing taking lithium should take a reduced dosage or even stop taking lithium in the critical period of heart development (4 to 12 weeks). However, lithium should never be discontinued abruptly. An increase in the GFR and an expansion of the blood volume occurring during pregnancy can cause a decrease in lithium levels during the first and second trimesters (66). Therefore, the lithium dosage may have to be increased in order to ensure sufficient lithium levels (67). Most guidelines propose to check lithium levels every 2 to 4 weeks, for the last trimester a weekly check is recommended (63, 66, 68, 69). Further monitoring is needed if patients develop preeclampsia, hyperemesis gravidarum, or suffer from fever. During the first trimester and concurrent lithium treatment, foetal ultrasound should be performed to check for cardiac malformations (63, 70, 71).

### Lithium and Breastfeeding

There is a controversial debate on whether or not lithium should be continued during breastfeeding, since studies investigating its effects on the child are scarce (70). As there is an increased chance of a recurrence of BD with lithium discontinuation in general (57) and during the postpartum period in particular, a change of treatment regimen for breastfeeding is not the first choice. In support of this, Bergink (72) could verify that patients with a new episode of BD during postpartum taking lithium had fewer recurrences after 9-month postpartum compared to patients receiving an antipsychotic treatment. Most of the guidelines conclude with a negative benefit-risk ratio for breastfeeding during lithium treatment (63, 70).

## Lithium in Older Age Bipolar Disorder

Patients aged 60 years and older account for a quarter of all BD patients (73). Nonetheless, pharmacological and non-pharmacological treatment strategies in these individuals are hardly studied in RCTs (74). BD patients in older age have a significantly reduced life expectancy of up to 15 to 20 years. They are at a higher risk of suffering from metabolic syndrome, cardiovascular diseases, general renal diseases and cognitive decline (75–77) compared to the general population. The lower life expectancy means that older patients may be exposed to prophylactic medication for a shorter period of time than younger patients, potentially translating to a reduced risk of long-term side effects (e.g., with regard to kidney function). Based on the available data, there are treatment recommendations for BD in older age in international treatment guidelines (31).

Lithium is considered the first line medication in older aged patients with BD in the maintenance treatment. It is recommended for the prevention of depression and mania given the evidence from adult studies, but also because the largest number of studies has been conducted for lithium in geriatric patients (73). However, due to concerns regarding tolerability and adverse events, lithium is prescribed less frequently in older individuals, although it is usually well tolerated by most of these patients (78, 79).

The randomized controlled GERI−BD trial investigated the efficacy and tolerability of lithium and divalproex in the treatment of mania in older age (60 years and above). Target serum concentrations were 0.80–0.99 mEq/L for lithium and 80–99 μm/L for valproate.

In this trial, lithium and valproic acid demonstrated to be equally effective in the 3-week follow-up (74) but lithium was more effective in reducing manic symptoms after the 9-week follow-up (74). Lithium and valproate showed a comparable frequency of side effects and were generally tolerated, providing evidence for lithium and valproate to be relatively safe with few adverse effects in BD in older adults.

### Special Considerations for Using Lithium in Older Age

It is recommended that therapeutic lithium levels should be lower in patients with BD and older age (31, 80, 81). Dehydration is common in older patients and should be prevented, particularly if lithium is prescribed. Special attention should be paid, if other substances are prescribed that carry a risk of increasing lithium blood levels. Medications that should be prescribed carefully under these circumstances are diuretics, angiotensin-converting enzyme (ACE) inhibitors, angiotensin receptor blockers (ARBs), but also non-steroidal anti-inflammatory drugs (NSAIDs). Lithium blood levels and renal parameters should be checked regularly, especially at the beginning of the treatment (82, 83).

The recommended lithium levels in BD patients in older age are 0.4–0.8 mmol/L (31). For depressive episodes and maintenance therapy, serum concentrations are recommended to be low as 0.4–0.6 mmol/L (9). The recommended serum levels for the treatment of mania are indicated at 0.4–0.8 mmol/L for ages 60–79 years and a bit lower (0.4–0.7 mmol/L) for patients with the age of 80 and above (80). This is reasonable as lithium levels higher than 0.8 mmol/L may cause neurocognitive and neurological symptoms (84) and renal side effects (85). Long-term treatment with lithium is associated with a reduction of the GFR and a twofold increased risk of chronic kidney disease (86). Patients with BD in older age are consequently at a higher risk of these adverse effects after having taken lithium for several years (78). Lithium levels and renal function should therefore be controlled every 3 months in patients aged 65 and above.

## Side Effects of Lithium Over the Lifespan

Lithium treatment is associated with a number of undesired side-effects that involve different organ systems. Gastro-intestinal complaints such as nausea, vomiting, and diarrhoea are relatively common. Nausea affects around 10–20% of patients, particularly in the early phase of treatment and often diminishes with longer treatment duration (87). Fine hand tremor is reported by about 25% of patients treated with lithium (88), the absolute risk increase was 9.6% compared to placebo in short-term trials [NNH = 10, (89)]. Clinically, it presents as postural tremor and its frequency and intensity correlate with lithium serum levels (90). Management mainly includes dose reduction. Pharmacological management with propranolol is recommended only in cases of severe tremor, as beta-blockers may introduce other side-effects (91).

The risk of hypothyroidism is increased 5.78-fold compared to placebo (92) and lithium may lead to increased blood calcium and parathyroid hormone levels (93). TSH and calcium levels should therefore be monitored regularly; hypothyroidism can be managed by levothyroxine substitution and is no reason for discontinuing lithium.

Cardiological harms may also arise from lithium treatment. In a recent review, the most common ECG abnormality was T wave inversion. Further alterations include QT prolongation and ventricular tachyarrhythmias (94).

Renal complications are another important concern in lithium-treated patients. A reduced concentration ability of the kidneys is associated with longer treatment duration (95) and is found in 54% to 73% of patients (95, 96). With lithium treatment, an excess decline of 15% in the kidney's ability to concentrate urine is found compared to untreated controls (92). However, as these results stem from observational data, they have to be interpreted with caution. A reduced concentration ability can manifest as a nephrogenic diabetes insipidus (NDI), clinically characterized by polyuria and polydipsia. Patients with NDI are at higher risk of lithium intoxication and should therefore be monitored more closely (97). Early detection is recommended and involves the assessment of “urine volume, frequency of urination, nycturia, thirst, and fluid intake”. Several treatment options have been suggested with limited evidence (97). In almost all cases, NDI is reversible when lithium is stopped.

With long-term use, lithium can cause chronic tubulo-interstitial nephritis, which is characterized by a decrease in the glomerular filtration rate (GFR) and may lead to chronic kidney disease (lithium nephropathy) (97, 98). While a detrimental effect of lithium on the kidneys seems certain, it remains unclear to which extend the observed decline in GFR is due to age-related renal impairment. A reduction in GFR of 0–5 ml/min is seen over 1 year and the risk of renal failure has been estimated to be about 0.5% compared to 0.2% in controls (92). A clinically relevant impairment of the renal function does usually not occur before at least 15 years of lithium treatment (99). Due to a lack of long-term studies, the evidence on lithium nephropathy is scarce and no clear recommendations regarding its management exist (97). If the kidney function is impaired, reducing the dosage or stopping lithium treatment may be necessary. Unlike NDI, renal failure may not be reversible upon lithium discontinuation.

A potential negative effect of lithium on cognition has been debated for many years. Typical complaints of lithium-treated individuals involve lethargy, fatigue, lack of mental clarity, and an inability to concentrate (100). Cognitive side effects may be a reason for non-adherence to lithium treatment (101). While BD is associated with cognitive dysfunctions, it remains unclear, to which extend these arise due to the illness or the treatment. A recent narrative review concluded that lithium may improve some cognitive functions while deteriorating others (102). Lithium negatively affects “immediate verbal learning and memory (SMD 0.29, 95% CI 0.07 to 0.51), creativity (SMD 0.34, 95% CI 0.00 to 0.68) and psychomotor performance (SMD 0.62, 95% CI 0.27 to 0.97)”, although these findings are based on relatively few patients (103). Lithium-induced hypothyroidism may be partly responsible for these cognitive deficits that can thus be counter-acted by levothyroxine substitution (104). In contrast, evidence from preclinical (105, 106) and clinical research (107) suggests that lithium may have beneficial effects on the incidence and progression of dementia. In support of this, a large population-based study found a negative correlation between lithium intake and dementia incidence (108). Patients with BD have a higher risk of developing dementia compared to the general population and lithium may be protective in these patients (109).These findings are based mainly on correlational data, however, and thus need confirmation by randomized trials.

A well-known and often unpleasant side-effect is weight gain. It is seen more often than with placebo (OR 1.89, 95% CI 1.27 to 2.82, p=0.002). Bowden et al. reported an average increase in body weight of 6 kg after a treatment duration of 1 year. Interestingly, weight gain was only observed in patients that already were obese at the beginning of the study. Among non-obese people, no such weight gain was observed (110).

Serum levels of lithium have to be closely monitored to avoid lithium intoxication, particularly in the elderly. Lithium intoxication is characterized by confusion, gross tremor (as opposed to a fine tremor as a typical side effect with therapeutic serum levels), ataxia, falls, convulsions, and gastrointestinal symptoms such as vomiting and diarrhoea. A retrospective cohort study found it to occur in around 1 in 100 person-years (111). Among 1,340 patients that were followed for 17 years, no deaths due to lithium intoxication were reported. Management includes stopping lithium, giving water (per os or parenterally), haemodialysis, and forced diuresis (111).

There are a few contraindications that need to be considered before initiating lithium treatment. These include cardiac pre-conditions such as certain types of arrythmias (e.g., Brugada syndrome), previous or current myocardial infarction and kidney failure.

## Lithium and Suicidality

The life expectancy of patients with BD is reduced by about 10 years compared to the general population (112) and the mortality gap seems to be increasing rather than decreasing over the years (113). A major cause of death is suicide, which is around 20–30-fold more frequent than in the general population (114). Around 25–50% of patients with BD attempt suicide at some point in their life and around 15% of patients die of suicide (115). The prevention of suicides is therefore a pivotal goal in the treatment of BD.

Lithium has been associated with a reduced suicide risk in patients with affective disorders including BD in a number of studies with varying methodology (116–118). Long-term studies suggest a strong suicide-preventing effect with suicides being 82% less frequent during lithium treatment (119). The studies supporting this suicide-preventing effect often include observational data, however, which are prone to bias. Adherence to lithium treatment and lower risk for suicidal behavior may correlate spuriously and may at least in part not be causally related (120). Lithium treatment is likely to be introduced when patients are at their worst, followed by a period of improvement. The increase in suicide risk after discontinuation of lithium treatment may be due to rebound depression or withdrawal effects. Additionally, patients may stop their lithium medication because their health is deteriorating, while continuously adhering to their medication when they are doing well.

More reliable evidence comes from RCTs. The largest meta-analysis investigating lithium and suicidality in RCTs found a reduction in suicides from 6/241 in the placebo group to 0/244 in the lithium group, corresponding to an absolute risk reduction of 2.5% [NNT = 40, (3)]. Only one trial did not use a withdrawal design, which may introduce bias due to withdrawal effects in the placebo group. This 1-year trial reported three suicides in the placebo arm and 0 suicides in the lithium arm, making the estimate more uncertain (121)

A relationship between lithium concentrations in tap water and a reduced suicide risk has even been postulated multiple times (122), which a more recent study did not replicate (122). The mechanism by which lithium may reduce suicides is hypothesized to be the reduction of impulsive and aggressive behavior in bipolar and depressed patients. By some authors, it is thought to have a specific anti-suicide effect exceeding its mood stabilizing properties (123).

As suicides are a special case of death, all-cause mortality seems a more relevant outcome, unless deaths of other causes (such as due to lithium intoxication or kidney failure) are favored over suicides. Investigating this, Cipriani et al. included eight RCTs with 782 patients and found a reduction in all-cause mortality in the lithium group of 2.31% over an average of around 81.5 weeks (Absolute risk: 5/392 (1.28%) vs. 14/392 (3.59%), absolute risk reduction: 2.31%, corresponding to an NNT = 43). This risk reduction was rather uncertain, however, with a wide 95% confidence interval for the odds ratio of 0.15 to 0.95.

It is possible that the mortality rate of patients treated with lithium is not constant over time. Specifically, lithium-induced deaths due to harms such as kidney failure may result after year-long exposures, thereby possibly reducing the overall benefit on mortality.

As the absolute risk reduction is rather small, it seems uncertain, if lithium reduces overall mortality over a course of years, which is the usual duration of treatment.

## Discontinuation of Lithium

Psychiatric patients, including patients with BD, often stop medication, e.g., due to concepts of their illness and treatment that differ from those of their physicians. A lack of insight into being ill is another reason. Furthermore, as lithium treatment is associated with adverse effects and the long-term effects on the body are insufficiently understood, many patients stop their lithium medication due to unwanted effects. The most common reasons are diarrhoea, tremor, diabetes insipidus, creatinine increase, and weight gain (124). Furthermore, lithium nephropathy can make discontinuation of lithium necessary.

Abrupt lithium discontinuation in patients stabilized on the medication is associated with severe adverse effects, such as suicidal behavior (59). In a study investigating this issue, 3 of 18 patients relapsed within 4 days after discontinuation, two of which had been stable on lithium for as long as 42 and 58 months (125). In a study of 64 patients with BD that were investigated in a naturalistic setting, the relapse risk increased from 53.3% to 94.1% (NNH = 2.5) from gradual (2–4 weeks) to rapid (< 2 weeks) discontinuation over 5 years (126). Similar results were found in a randomized trial of 161 bipolar patients. Abrupt discontinuation (1–14 days) led to a 20-fold increase in relapse rates as compared to gradual reduction (15–30 days) over a course of 3 years (37% vs. 1.8%, p < 0.0001, (59)). The median time to recurrence was decreased from 14.0 months to 2.5 months (127). The same pattern of increased relapse rates is seen in pregnant women (128).

The risk of relapse after abrupt discontinuation seems to exceed the risk before starting lithium treatment and may thus be considered an iatrogenic harm (rebound phenomenon, (129). It is therefore strongly recommended to discontinue lithium very slowly. Whether a gradual withdrawal over more than 4 weeks is more beneficial has not been investigated to date.

It is likely that rebound after discontinuation undermines the validity of relapse prevention trials, in which stabilized patients are withdrawn from lithium within a few weeks before being randomized to placebo (129). The benefit of lithium could thus be smaller than generally assumed.

If lithium discontinuation is followed by a relapse, patients may want to reinstate lithium medication. Reintroduction of lithium after discontinuation does not seem to affect treatment efficacy (130), as had been speculated by some authors in the past (131).

## Conclusions

BD is a debilitating illness with an often chronic clinical course that usually requires lifelong treatment. Lithium has been used for treating BD for many years since the discovery of its antimanic properties in 1949 (132). Notwithstanding the introduction of other therapeutic agents that have led to a reduction in prescription of lithium in the past decades (133), it still remains the gold standard in the treatment of BD, particularly over the lifespan with the highest efficacy in the maintenance treatment. The proposed suicide-preventing effect of lithium is unique, while possibly being less substantiated than previously thought. It is an efficacious medication in the acute treatment of mania, while lacking efficacy in the treatment of bipolar depression. Lithium can be used for the treatment of BD in all age groups. It is effective in paediatric BD and can be used, with caution, during pregnancy. Lithium use is generally not recommended during breastfeeding, however. Older aged patients with BD also benefit from lithium, while the serum levels are recommended to be rather lower than in younger adults. Lithium is generally well tolerated. However, adverse events, such as hypothyroidism, renal dysfunctions, and cognitive side effects have to be weighed against the benefits. Special care is strongly recommended when discontinuing lithium, as rapid withdrawal may lead to severe side-effects.

In sum, lithium remains an effective and generally tolerable therapeutic agent for BD. Every clinically active psychiatrist should be able to handle lithium comfortably, such that the harms are minimized and the benefits outweigh the risks. We hope that this review helps guide practitioners and patients alike.

## Author Contributions

CV and SK drafted and wrote the manuscript. TB provided a critical revision of the article.

## Funding

We acknowledge support from the German Research Foundation (DFG) and the Open Access Publication Fund of Charité – Universitätsmedizin Berlin.

## Conflict of Interest

The authors declare that the research was conducted in the absence of any commercial or financial relationships that could be construed as a potential conflict of interest.
